# Reduction in Nitrogen Rate and Improvement of Nitrogen Use Efficiency without Loss of Peanut Yield by Regional Mean Optimal Rate of Chemical Fertilizer Based on a Multi-Site Field Experiment in the North China Plain

**DOI:** 10.3390/plants12061326

**Published:** 2023-03-15

**Authors:** Jiayu Hu, Yang Yang, Hongyan Zhang, Yuhao Li, Shuhong Zhang, Xinhua He, Yufang Huang, Youliang Ye, Yanan Zhao, Jungying Yan

**Affiliations:** 1 College of Resource and Environment, Henan Agricultural University, Zhengzhou 450002, China; 2 Innovation Centre for Efficient Use of Nitrogen Fertilizer, Henan Xinlianxin Chemical Industry Group Co., Ltd., Xinxiang 453700, China; 3 Centre of Excellence for Soil Biology, College of Resources and Environment, Southwest University, Chongqing 400715, China; 4 School of Biological Sciences, University of Western Australia, Perth 6009, Australia; 5 Henan Province Soil and Fertilizer Station, Zhengzhou 450002, China

**Keywords:** *Arachis hypogaea* L., fertilizer use efficiency, nutrient uptake, optimal fertilization, pod yield

## Abstract

It is important to quantify nutrient requirements and optimize fertilization to improve peanut yield and fertilizer use efficiency. In this study, a multi-site field trial was conducted from 2020 to 2021 in the North China Plain to estimate nitrogen (N), phosphorus (P), and potassium (K) uptake and requirements of peanuts, and to evaluate the effects of fertilization recommendations from the regional mean optimal rate (RMOR) on dry matter, pod yield, nutrient uptake, and fertilizer use efficiency. Results show that compared with farmer practice fertilization (FP), optimal fertilization (OPT) based on the RMOR increased peanut dry matter by 6.6% and pod yield by 10.9%. The average uptake rates of N, P, and K were 214.3, 23.3, and 78.4 kg/ha, respectively, with 76.0% N harvest index, 59.8% P harvest index, and 41.4% K harvest index. The OPT treatment increased N, P, and K uptake by 19.3%, 7.3%, and 11.0% compared with FP, respectively. However, the average of yield, nutrition uptake, and harvest indexes of N, P, and K were not significantly affected by fertilization. The peanut required 42.0 kg N, 4.6 kg P, and 15.3 kg K to produce 1000 kg of pods. The OPT treatment significantly improved the N partial factor productivity and N uptake efficiency but decreased the K partial factor productivity and K uptake efficiency. The present study demonstrates that fertilizer recommendations from RMOR improve N use efficiency, and reduce N and P fertilizer application without yield loss in regions with smallholder farmers, and the corresponding estimation of nutrient requirements helps to make peanut fertilization recommendations.

## 1. Introduction

Peanut is an important oilseed crop, an N_2_-fixing legume plant with drought tolerance, and a very efficient cash crop with relatively low production input, high yield, and higher price and greater income than other oil crops [1]. The extraction rate of peanut oil is high, and the oil quality is good. Therefore, peanut plantations play an important role in meeting edible oil demand and supporting local economies. China is a major peanut producer, ranking second in planting area after India and first in total production [2]. In 2020, the planting area reached five million hectares, and the total production reached 18 million tons, accounting for more than 20% and 40%, respectively, of the global totals [2]. Nitrogen (N), phosphorus (P), and potassium (K) are essential nutrients for crops. Application of NPK fertilizer can improve crop yield [3].

Although peanut is a legume with the ability to fix atmosphere N, its growth requires adequate N, P, and K nutrients by fertilization [3]. The N fixation by rhizobia can meet 40% to 50% of the N requirement of peanut plants [4]. However, the N fixation level by peanut rhizobia is significantly and negatively correlated with increasing N application [4]. China is the world’s largest consumer of chemical fertilizer [5]. In recent years, unreasonable fertilization in pursuit of higher crop yields has become common, while yield response to fertilization has become lower. Unreasonable fertilizer application not only increases production costs and reduces FUE but also causes environmental pollution [5,6]. Therefore, it is an important challenge for sustainable peanut production to ensure high peanut yield, quality, and FUE through rational fertilization.

In recent decades, studies have attempted to develop various methods to optimize the application rates of chemical fertilizer to solve the associated economic and environmental problems caused by improper application. These techniques of fertilization recommendation were based on soil testing [7], aboveground plant analysis [8], yield–fertilizer rate response modeling [9], digital and information technology [10,11,12], and integrated soil-crop system management [13,14]. Most of these recommended techniques have focused on site-specific optimal nutrient rates and have required field trials, soil samples, plant collection, and laboratory analysis, factors that can be rather time-consuming, labor-intensive, and expensive. Although these methods are useful for precision fertilization, they are rather difficult to implement widely for many smallholders in developing countries including China [15]. Firstly, arable land in these countries/regions is limited, with small sizes and scattered field plots. Secondly, in the rural regions of these areas, there is a lack of testing conditions and technical guidance for agrochemical services, with insufficient testing equipment and technical personnel. Finally, these field sites are highly intensive with two or sometimes three crops planted annually in the same field, and testing is difficult to perform in time to avoid delaying planting.

In the practice of agricultural production, it is critical to establish a simple method for the overall regulation and promotion of the rational application of fertilizer on a regional scale rather than a field scale [16]. Numerous factors affect crop yields, and some are difficult to predict accurately. Therefore, even for the same field, the suitable fertilizer application rate and related parameters obtained from an experiment in one year may not be accurately applied to other years [17]. Accordingly, a very high degree of accuracy is unnecessary to calculate the appropriate fertilizer application rate for obtaining a certain yield. For agricultural production, the determination of a suitable fertilizer application range can basically meet the needs of large-scale production.

A regional mean optimal rate (RMOR) of N fertilizer application has been proposed to resolve this technology gap in Tai Lake Region, China [18]. The purpose of the resolution is to provide a baseline fertilization recommendation for a specific region by determining the RMOR of this region to obtain the maximum total production and economic benefits. The RMOR can be the average of the optimal fertilizer rate obtained from the multi-site trial, and is further used as the fertilization recommendation for the whole region [18]. However, the optimal fertilizer rate can be determined by other methods, such as nutrient requirement to produce unit yield. The method has been proven feasible for crops such as rice, wheat, and oilseed rape; the recommendations have produced increasing yields, income, and nutrient use efficiency and have reduced environmental impacts [19,20]. Adjusting the RMOR for different regions, crop types, and soil conditions can provide a more accurate estimation of the optimal rate of fertilizer applied. However, the effectiveness of this resolution in other regions, crop types (N-fixing crops such as peanut), and nutrients (P and K fertilizer) is unknown.

Here, a two-year period (2020 and 2021) of multi-site field experiments were conducted in the largest peanut-growing area of the North China Plain. The hypothesis is that the ROMR method can maintain peanut yield and FUE while reducing the chemical fertilizer at a regional scale. The overall goals of this study were to quantify (1) N, P, and K uptake and requirements; (2) effects of the RMOR on peanut yield, nutrient uptake, and utilization efficiency; and hence (3) effectiveness of this RMOR method in peanut farming at the county scale.

## 2. Materials and Methods

### 2.1. Experimental Site

**Figure 1:**
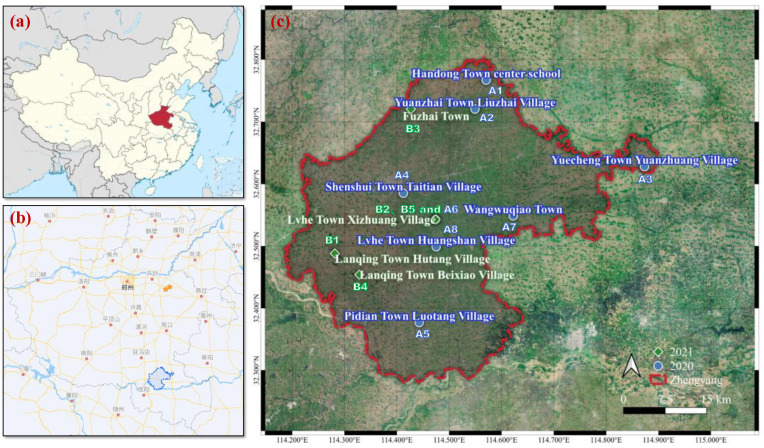
The geographical locations of experimental field sites. (**a**) Henan Province is shown as a red background in the China map; (**b**) the blue border is the Zhengyang County; (**c**) distribution of experimental field sites in the Zhengyang County.

**Figure 2:**
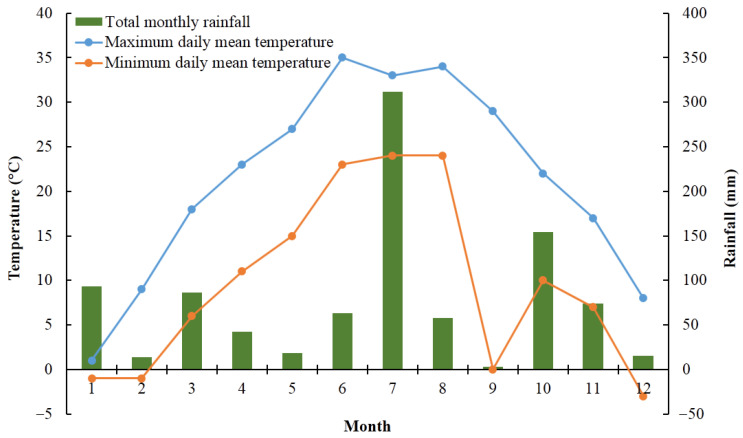
The monthly temperature and rainfall in Zhengyang County.

The field experiments were conducted in 2020 and 2021 in several townships in Zhengyang County (N32°16’-32°47’, E114°12′-114°53′), Henan, China (Figure 1). Both the peanut planting area and production of Zhengyang county rank first among all counties in China. The area is located in the transition region from the north subtropics to the warm temperate zone, and it has a continental monsoon humid climate. The average annual temperature is 15.3 °C, and the average annual precipitation is 935 mm. The monthly temperature and rainfall are shown in Figure 2 [21].

### 2.2. Field Experiment Design

There were eight field sites in 2020 and five sites in 2021 for the experiment (Figure 1). The planting pattern was summer peanut and winter wheat rotation. The soil type was Alfisol according to the Soil Taxonomy with clay texture and low pH value. The basic soil physiochemical properties are shown in Table 1.

Each field experiment was set up with two treatments, farmers’ practice fertilization (FP) and optimized fertilization (OPT). All management of the FP treatment was determined by farmers based on their practices, and the rate of fertilizer applied was accurately recorded (Table 1). As a management strategy focused technology promotion, the determination of RMOR must be based on other approaches or data. Therefore, for the OPT treatment, the RMOR was determined based on the results of previous studies with similar environment, in which, the optimized amount of fertilizer was determined according to the yield–fertilizer rate response modeling [22]. In brief, a total of 46 field trials including four NPK fertilizer rates were collected. The average value of yield and fertilizer application under the same treatment was obtained. The yield–fertilizer rate response modeling was simulated by quadratic equation (y = ax^2^ + bx + c, y: yield; x: N, P, or K fertilizer rates). Then the RMOR for NPK fertilizer was calculated at the maximum yield according to the modeling (-b/2a). The fertilizer recommendations were further adjusted by nutrient experts based on local conditions. The N, P, and K fertilizer rates for OPT in 2020 and 2021 were 181.5 kg/ha, 39.3 kg/ha, and 93.4 kg/ha, respectively (Figure S1). The other management measures were completely consistent with the FP treatment. Each treatment was repeated three times in plots of greater than 100 m^2^.

All peanuts were sown in early June and harvested at the end of September. The peanut varieties were decided by the farmers themselves, and they all used the widely grown varieties of momordica fruit type. Before peanut sowing, soil was plowed and then tilled. The peanuts were planted in ridges with a width of 80 cm at the bottom and 60 cm at the surface. Two rows of peanuts were planted on the ridge surface with a spacing of 20 cm. The overall sowing rate was 225 kg/ha. Ridging, sowing, and fertilizing were completed by mechanized methods performed once. Other management such as weed, pest, and disease control was consistent with FP treatment.

### 2.3. Sampling and Measurement

At peanut maturity, 2 m^2^ peanut samples were taken for yield measurement in each plot of all experiment sites. The air-dried sample was weighed and its moisture content was determined, and further converted to the weight with a moisture content of 8%. Meanwhile, five representative plants were taken at random for dry matter (DM) and nutrient measurement. The haulm and pod were cleaned and sterilized at 105 °C for 30 min, then dried at 75 °C. After the plant and pod were dried to a constant weight, the DM weight (plant and pod) was recorded. The dried samples were crushed and passed through a 2 mm sieve for the determination of N, P, and K contents. A 0.2 g sample was digested with H_2_SO_4_-H_2_O_2_ to obtain a solution. The total N and P contents were determined using a flow injection analyzer (AA3, Seal, Germany), and the K content was determined by a flame photometer [23]. The fatty acid composition of peanut seeds was measured by the gas chromatography.

### 2.4. Data Processing and Analysis

Taking N as an example, parameters of NHI (N harvest index), RIE_N_ (N reciprocal internal efficiency), PFP_N_ (N fertilizer partial factor productivity), NUpE (N uptake efficiency), and NUtE (N utilization efficiency) were used to evaluate the characteristics of nutrient uptake and utilization [24,25]. The parameters were calculated as follows:

NHI = N uptake in pod/total N uptake (1)

RIE_N_ = total N uptake/pod yield × 1000 (2)

PFP_N_ = pod yield/N fertilizer rate (3)

NUpE = total N uptake/N fertilizer rate (4)

NUtE = pod yield/total N uptake (5)

The data were processed by Microsoft Excel 2016 software and graphed by Origin Lab Origin 2018 software. The statistical analysis of data were performed in SPSS 20.0 software. A one-way ANOVA was used for significant tests at *p* < 0.05.

## 3. Results

### 3.1. Dry Matter (DM), Pod Yield (PY), and Harvest Index (HI)

**Figure 3:**
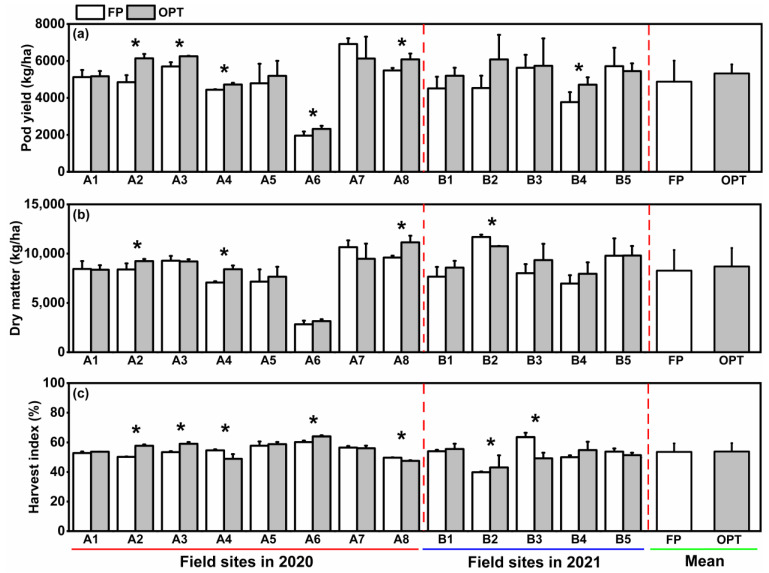
Variations in dry matter (DM, (**a**)), pod yield (PY, (**b**)), and harvest index (HI, (**c**)) of different fertilization treatment at different field sites in 2020 and 2021. FP: farmers’ practice fertilization; OPT: optimized fertilization. A: experiment in 2021; B: experiment in 2022. Tentacle lines on the bars are standard deviation. Asterisks (*) on the bars indicate a significant difference at *p* < 0.05 between fertilization treatments for the same field site.

The PY ranged from 1958 kg/ha to 6915 kg/ha and averaged 4879 kg/ha for 13 field sites in 2 years. Compared with the FP treatment, OPT significantly increased PY in six field sites (Figure 3a). The yield increases by the OPT treatment ranged from -784 kg/ha to 1549 kg/ha and averaged 10.9%. The response of DM to different treatments was consistent with PY at 13 sites over 2 years (Figure 3b). Compared with FP, the DM increase in OPT ranged from −1179 kg/ha to 1544 kg/ha with an average rate of 6.6%. The variation range of peanut HI was 39.9%–64.0% with an average of 54.0% (Figure 3c). The peanut HI under OPT increased significantly in three field sites while decreasing significantly in other three field sites. There was no significant change in HI in total between FP and OPT treatments.

### 3.2. N, P, and K Uptake

**Figure 4:**
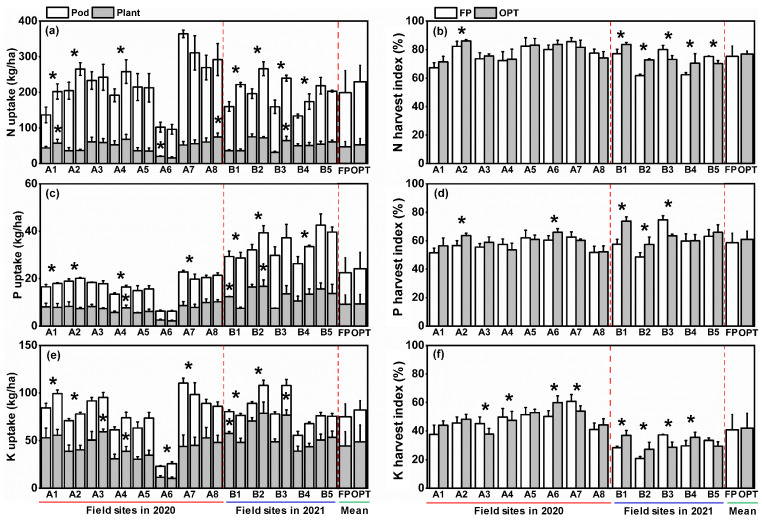
Uptake and nutrient harvest indexes of N (**a**,**b**), P (**c**,**d**), and K (**e**,**f**) in peanuts under different fertilization treatments. FP: farmers’ practice fertilization; OPT: optimized fertilization. A: experiment in 2021; B: experiment in 2022. Tentacle lines on the bars are standard deviation. Asterisks (*) on the bars indicate a significant difference at *p* < 0.05 between fertilization treatments for the same field site.

There was significant variation in peanut nutrient uptake at different sites (Figure 4). For the two years, total N, P, and K uptake rates were 96.3–364.2, 6.2–42.6, and 23.0–110.3 kg/ha, and averaged 214.3, 23.3, and 78.4 kg/ha, respectively (Figure 4a,c,e). The total N, P, and K uptake rates under OPT were generally higher than in the FP treatment, with increasing rates of 19.3%, 7.3%, and 11.0%, respectively. In general, the N and P uptake rates by peanut straw were lower than for pods, while the K uptake was higher by straw than by pods, with a lower KHI (Figure 4f). The N, P, and K uptake rates were 164.3, 14.0, and 31.8 kg/ha in pods, and 50.0, 9.3, and 46.6 kg/ha in straw, respectively. For these 2 years, the NHI, PHI, and KHI were 61.7%-86.1%, 48.6%-74.7%, and 20.9%-60.7%, respectively, and averaged 76.0%, 59.8%, and 41.4%, respectively (Figure 4b,d,f). Although there were differences between FP and OPT in several field sites, in general, the OPT treatment did not significantly affect nutrient harvest indexes of peanuts in 13 field sites.

### 3.3. Reciprocal Internal Efficiency (RIE)

**Figure 5:**
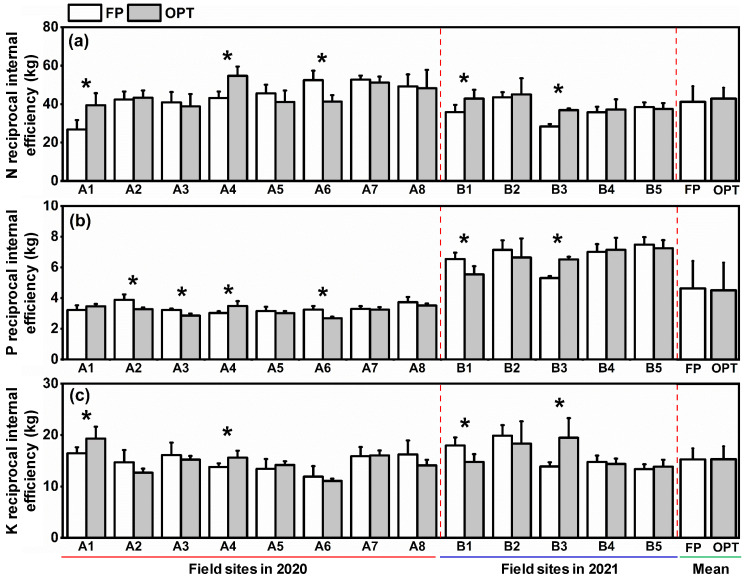
Requirements of N (**a**), P (**b**), and K (**c**) to produce 1000 kg pod yield of peanuts (reciprocal internal efficiency (RIE) under different treatments. FP: farmers’ practice fertilization; OPT: optimized fertilization. A: experiment in 2021; B: experiment in 2022. Tentacle lines on the bars are standard deviation. Asterisks (*) on the bars indicate a significant difference at *p* < 0.05 between fertilization treatments for the same field site.

For 13 field sites in the 2 years, the RIE of N (RIE_N_) averaged 42.0 kg with a range of 26.8–54.7 kg (Figure 5a). The averaged RIE_N_ was 6.9% higher under OPT than under FP. The RIE of P (RIE_P_) in the 2 years varied from 2.7 kg to 7.5 kg with an average of 4.6 kg (Figure 5b). The RIE of K (RIE_K_) varied from 11.1 kg to 19.9 kg with an average of 15.3 kg (Figure 5c). There was no significant difference in averaged RIE_K_ or RIE_P_ between FP and OPT treatments.

### 3.4. Partial Factor Productivity of NPK Fertilizer

**Figure 6:**
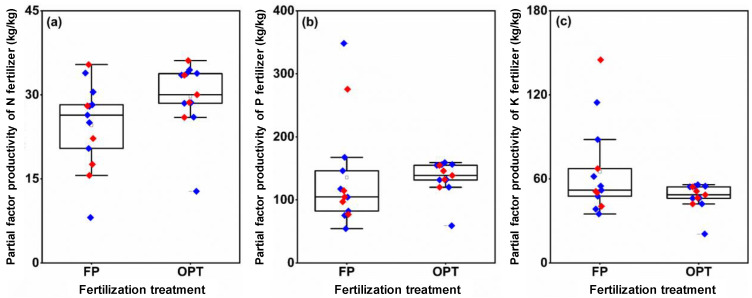
Partial factor productivity of N (**a**), P (**b**), and K fertilizer (**c**) under different fertilization treatments. FP: farmers’ practice fertilization; OPT: optimized fertilization. For each boxplot, the top, middle, and bottom solid lines of the box represent the 75% quantile, 50% quantile, and 25% quantile, respectively; the top and bottom horizontal lines outside the box represent the 90% quantile, and 10% quantile, respectively. The square inside the box represents the mean. Blue or red diamonds: data in 2020 or 2021.

Compared with the FP treatment, the average PFP_N_ of OPT increased by 15.1% in 2020 and by 29.8% in 2021 (Figure 6a). The average PFP_P_ of the OPT treatment decreased by 2.4% in 2020 and increased by 3.4% in 2021 compared with FP (Figure 6b). However, the average PFP_K_ of OPT decreased by 23.7% and 31.5% in the 2 years, respectively (Figure 6c).

### 3.5. Nutrient Uptake Efficiency

**Figure 7:**
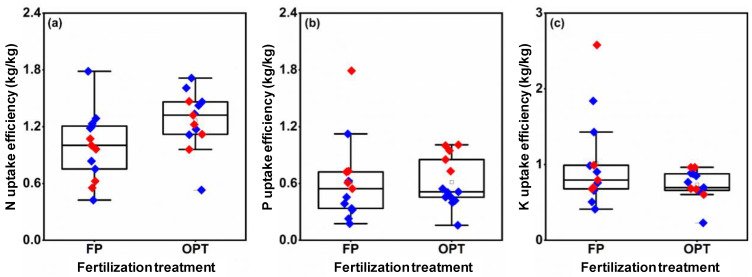
Uptake efficiency of N (**a**), P (**b**), and K (**c**) in peanuts under different fertilization treatments. FP: farmers’ practice fertilization; OPT: optimized fertilization. For each box plot, the top, middle, and bottom solid lines of the box represent the 75% quantile, 50% quantile, and 25% quantile, respectively; the top and bottom horizontal lines outside the box represent the 90% quantile, and 10% quantile, respectively. The square inside the box represents the mean. Blue or red diamonds: data in 2020 or 2021.

There was a significant variation in NUpE among different field sites (Figure 7a). From 2020 to 2021, the average NUpE of the OPT treatment was increased by 19.3% and 45.2% compared with FP. The average PUpE of OPT decreased by 6.5% in 2020 and increased by 3.4% in 2021 compared with FP (Figure 7b). However, the average KUpE of OPT decreased by 25.5% and 32.2% compared with FP (Figure 7c).

### 3.6. Nutrient Utilization Efficiency

**Figure 8:**
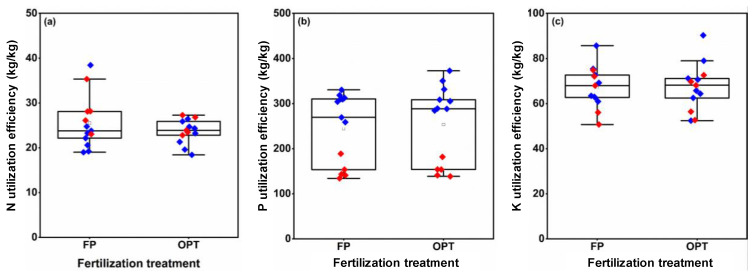
N (**a**), P (**b**), and K utilization efficiency (**c**) of peanuts under different fertilization treatments. FP: farmers’ practice fertilization; OPT: optimized fertilization. For each box plot, the top, middle, and bottom solid lines of the box represent the 75% quantile, 50% quantile, and 25% quantile, respectively; the top and bottom horizontal lines outside the box represent the 90% quantile, and 10% quantile, respectively. The square inside the box represents the mean. Blue or red diamonds: data in 2020 or 2021.

Compared with FP, the average NUtE for OPT was reduced by 3.8% in 2020 and by 11.7% in 2021 (Figure 8a). The average PUtE for OPT had a 4.8% increase in 2020 but an 11.7% decrease in 2021 compared with FP (Figure 8b). No clear difference in averaged KUtE between FP and OPT treatments was found in the 2 years (Figure 8c).

### 3.7. Fatty Acid Composition of Peanut Kernels

**Figure 9:**
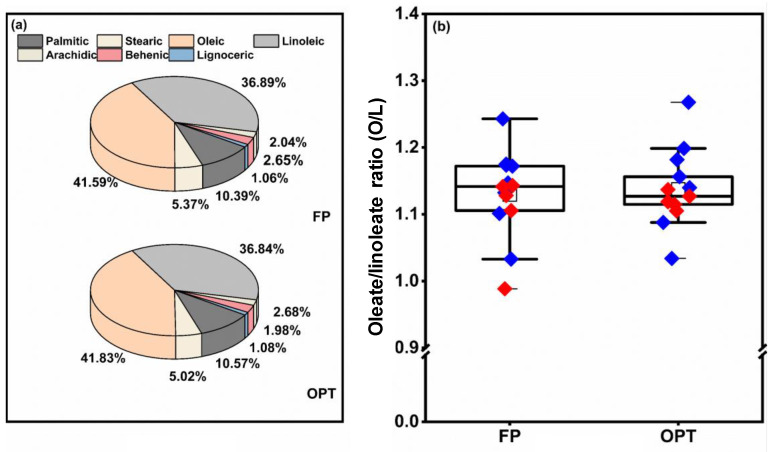
Fatty acid composition (**a**) and oleate/linoleate ratio (O/L, (**b**)) of peanut kernels under different fertilization treatments. FP: farmers’ practice fertilization; OPT: optimized fertilization. For each box plot, the top, middle, and bottom solid lines of the box represent the 75% quantile, 50% quantile, and 25% quantile, respectively; the top and bottom horizontal lines outside the box represent the 90% quantile, and 10% quantile, respectively. The square inside the box represents the mean. Blue or red diamonds: data in 2020 or 2021.

In terms of the fatty acid composition of peanut kernels, oleic and linoleic acids accounted for 42.1% and 36.7%, respectively, followed by palmitic acid and stearic acid at 10.7% and 5.0%, respectively (Figure 9a). The ratio of oleic acid to linoleic acid (O/L ratio) was only 1.2 (Figure 9b). In general, the OPT treatment did not significantly affect the fatty acid composition or O/L of peanut kernels compared with FP.

## 4. Discussion

### 4.1. Feasibility of Fertilizer Recommendation Based on RMOR

There are some common concerns in developing countries such as small and scatted field sites, lack of test conditions, and lack of technical guidance for agrochemical services [15,26]. The present study demonstrated that the unified fertilization recommendations (i.e., RMOR) can achieve a reduction in chemical fertilizer and N use efficiency without yield loss in peanuts for smallholders in country scale. In these regions, this method has high practical value; the maximum benefit can be obtained, and the fertilizer recommendation is easy to promote. The theoretical basis of the RMOR method is to optimize the fertilizer application in the region scale, thus improving soil condition, the growth characteristics, and nutrient utilization of peanuts [22,27]. In this study, the N and P fertilizer rates according to RMOR were lower than the application rate in peanuts by the farmer practice in this region. Therefore, the simplified method can also save on N and P fertilizer. This is of great practical significance for decreasing the chemical fertilization rate while improving FUE, and thus decreasing the environmental risk, especially in areas with excessive chemical fertilizer consumption [28]. Once the RMOR for a specific crop in a certain region is determined, it can be applied for several years as long as there are no significant changes in climate or cultivation conditions. Therefore, the RMOR can be used as a basis for fertilizer development planning in the region, and as a basis for recommended fertilizer application rates for the crop in different field sites.

### 4.2. Limitation and Uncertainty

The RMOR is a management strategy for the optimization of regional fertilization, rather than a calculations method. Therefore, the determination of RMOR for a specific region needs to be based on other methods [19]. Meanwhile, applying the RMOR in the entire region, the benefits for individual farmers vary in this study; for some field sites, yields and nutrient efficiency even decrease, though it would not result in a significant yield loss for the region as a whole. The appropriate fertilizer rate for a given crop is affected by diverse factors such as management, soil, climate, and tillage [26,29]. This study showed that even within the same county there was significant variation in peanut yield and nutrient requirement. Therefore, the RMOR and its control range when applied should be delimited according to different conditions in specific regions and field sites and should be determined again with the changes in production environments. Furthermore, the adverse effects of the simplified practice can be decreased when combined with the specific conditions of each field, and then one can make appropriate adjustments of increase or decrease such as according to the soil fertility level, organic fertilizer application, and previous stubble [19,30]. Especially, for counties and regions with high requirements for precision fertilization, adjustment according to the specific situation of the local field and/or the necessary testing is needed to avoid the loss of yield and economic benefits from individual field sites.

### 4.3. Nutrient Requirements of Peanut

Compared with other studies with large sample sizes, the N requirement to produce 1000 pods in this study was similar to the result of 42.2 kg [25], but slightly lower than in other studies [31,32]; P and K demand was lower than what was estimated across China [31] and in the same region of central north China [32]. Estimation variation in nutrient requirement may be related to differences in planting regions, cultivars, soil conditions, yield level, and tillage [30,33]. Crucsiol et al. [3] demonstrated that N absorption for older cultivars remains high, while newer cultivars were less demanding in N. Xie et al. [25] found that the values of nutrient requirement simulated by modeling were lower than the average observed values, and they explained that the N, P, and K predicted by the model were the optimal nutrient requirements under the conditions of the balanced absorption of N, P, and K. However, high soil nutrient supply and excessive fertilization practices may have resulted in excess nutrient uptake [31]. A large number of studies have demonstrated that crop nutrient uptake positively correlates with yield level, but the yield increase decreases when the yield reaches a certain level [32]. As a result, nutrient requirements per unit yield tended to decrease as yields increased, especially when yields were above 70% of the potential yield [25]. The peanut yield has been strongly promoted in the past few decades in China due to cultivar renewal, adequate fertilization, soil improvement, and other management changes [32]. This means that the nutrient uptake requirements to produce unit pods may be decreasing.

## 5. Conclusions

The results of multi-site peanut experiments supported the hypothesis that compared with the FP treatment, the OPT treatment based on the RMOR method promoted N use efficiency (PFP_N_ and NU_P_E), and decreased the nutrient inputs by chemical fertilizer, especially the N and P fertilizers, without the loss of peanut yield and NPK uptake. The NPK nutrient requirements of peanuts are quantified in this study, which is important for the regional recommendation of fertilization based on the RMOR. Therefore, the RMOR method is feasible for NPK fertilizer recommendations for peanut plantations, as it can simultaneously realize the optimization of agronomic, economic, and environmental benefits at a regional scale. The RMOR method can also be generally adopted in countries and regions with widespread smallholder farms. Furthermore, combined with precision fertilization technologies, the RMOR method is promising to realize agronomic and environmental optimization at the field scale.

## Figures and Tables

**Table 1:** Basic soil physiochemical properties and inputs of chemical fertilizers by the farmers’ practice (FP) at different field sites in 2020 and 2021.

Year	Field Site	Soil Property at 0–20 cm Depth						Nutrient Inputs by Chemical Fertilizer		
		pH	SOC (%)	NH_4_^+^-N (mg/kg)	NO_3_^-^-N (mg/kg)	Olsen-P (mg/kg)	NH_4_OAc-K (mg/kg)	N (kg/ha)	P (kg/ha)	K (kg/ha)
2020	A1	5.12	1.25	54.9	7.7	41.3	139.0	181.5	49.1	93.4
	A2	5.09	0.85	25.3	7.2	28.8	77.9	159.0	58.9	93.4
	A3	5.28	1.38	47.9	8.2	73.9	189.0	279.0	16.4	49.8
	A4	5.30	0.91	47.7	4.4	35.9	76.6	159.0	58.9	9.34
	A5	5.07	1.32	62.5	8.8	26.3	69.0	181.5	32.7	124.5
	A6	5.05	1.19	23.2	8.7	81.2	131.6	241.5	36.0	56.0
	A7	5.09	1.01	26.9	10.9	44.5	80.4	204.0	58.9	112.0
	A8	5.38	1.04	27.3	7.1	36.6	80.4	219.0	32.7	62.2
2021	B1	4.97	0.99	80.1	19.9	37.8	149.8	256.5	16.4	31.1
	B2	5.00	0.81	29.1	15.1	27.6	73.3	204.0	58.9	112.0
	B3	4.82	0.77	45.5	23.9	46.8	112.7	159.0	49.1	112.0
	B4	5.12	1.28	23.0	10.9	34.0	96.0	241.5	36.0	56.0
	B5	5.27	0.94	37.6	27.8	73.9	113.8	204.0	58.9	112.0
	Mean ± SD	5.12 ± 0.15	1.06 ± 0.21	40.8 ± 17.6	12.4 ± 7.0	45.3 ± 18.8	106.9 ± 36.7	206.9 ± 39.2	43.3 ± 16.0	85.2 ± 30.3

## Data Availability

The data presented in this study are available on request from the corresponding author.

## Supplementary Materials

The following supporting information can be downloaded at: https://www.mdpi.com/article/10.3390/plants12061326/s1, Figure S1: the yield-fertilizer rate response modeling for the RMOR of NPK fertilizer.
