# Supplementary material for: Reduction in Nitrogen Rate and Improvement of Nitrogen Use Efficiency without Loss of Peanut Yield by Regional Mean Optimal Rate of Chemical Fertilizer Based on a Multi-Site Field Experiment in the North China Plain
Source: Plants (Basel). 2023 Mar 15;12(6):1326. doi: 10.3390/plants12061326 (PMC10051281; doi:10.3390/plants12061326)

Figure S1. The yield–fertilizer rate response modeling for the RMOR of NPK fertilizer. In the quadratic equation, y represents pod yield of peanut; x represents N, P, or K fertilizer rates.

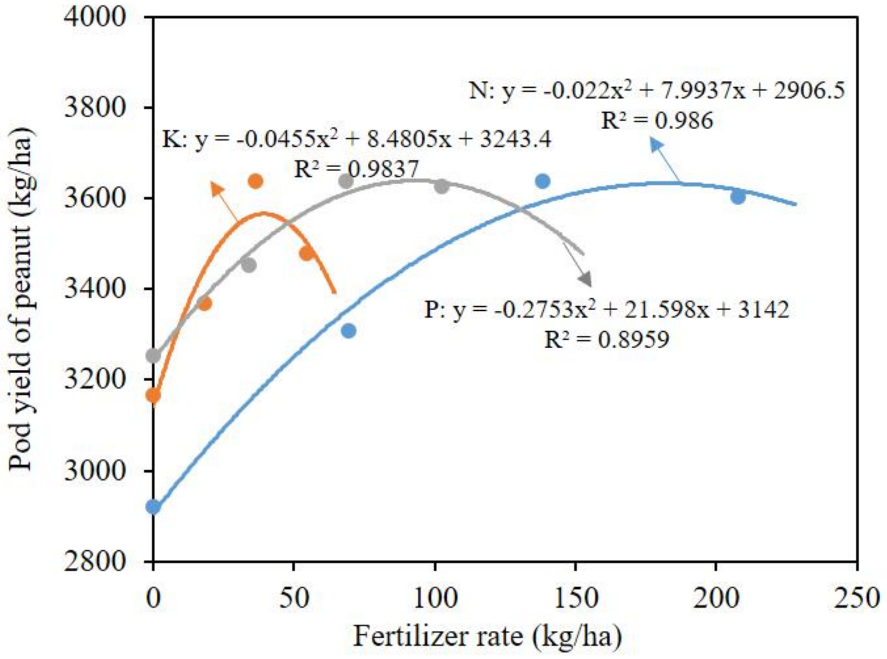

Supplement: Supplementary file 1 [file plants-12-01326-s001.zip › plants-2046769-supplementary.pdf]
